# The Effect of Anti-Viral Treatment of HCV Infection on Outcomes of Renal Transplant Patients with Chronic HCV Infection: A Real-World Cohort Study

**DOI:** 10.3390/biomedicines13112842

**Published:** 2025-11-20

**Authors:** Chih-Wei Chiu, Kuo-Ting Sun, Shih-Ting Huang, I-Kuan Wang, Chi-Yuan Li, Tung-Min Yu

**Affiliations:** 1 Division of Nephrology, Taichung Veterans General Hospital, Taichung 407, Taiwan; 2 Graduate Institute of Biomedical Sciences and School of Medicine, China Medical University, Taichung 404, Taiwan; 3 College of Medicine, China Medical University, Taichung 404, Taiwan; 4 Department of Post-Baccalaureate Medicine, College of Medicine, National Chung Hsing University, Taichung 402, Taiwan

**Keywords:** chronic hepatitis C virus, end-stage renal disease, anti-HCV viral infection treatment, direct-acting antivirals, kidney transplantation

## Abstract

**Background/Objectives:** Chronic hepatitis C virus (HCV) infection remains a significant comorbidity in patients with end-stage renal disease (ESRD), complicating outcomes after kidney transplantation. The anti-viral treatment of HCV infection including Direct-acting antivirals (DAAs) have transformed HCV treatment, but evidence remains limited. **Methods:** We conducted a retrospective, real-world cohort study using the TriNetX Analytics Network. Patients were divided into two cohorts: those who received anti-viral treatment of HCV infections before transplant (n = 982) and those who did not (n = 982), following 1:1 propensity score matching. **Results:** Outcomes assessed one year post-index included mortality, hepatic complications, graft failure, and serum creatinine >6 mg/dL. Anti-HCV infection treated patients had significantly lower risks of graft failure (aHR: 0.656; 95% CI: 0.434, 0.993; *p* < 0.001) and severe renal dysfunction (aHR: 0.619; 95% CI: 0. 0.390, 0.984; *p* < 0.001) compared to untreated patients. While mortality (aHR: 0.901; 95% CI: 0.728, 1.114) and liver-related outcomes trended favorably in the treated group, they did not reach statistical significance. **Conclusions:** Our findings demonstrate that pre-transplant anti-viral treatment of HCV infection in HCV-infected kidney transplant recipients is associated with improved graft survival and renal function.

## 1. Introduction

Chronic hepatitis C virus (HCV) infection, caused by a positive-sense RNA virus of the *Flaviviridae* family, genus *Hepacivirus*, results in significant comorbidity among patients with end-stage renal disease (ESRD). Historically, its presence has posed complex challenges in the field of kidney transplantation including adverse effects of graft survival, liver disease progression, and interaction with immunosuppressants [1]. Numerous studies have established that kidney transplantation offers superior long-term survival, quality of life, and cost-effectiveness compared to maintenance hemodialysis for ESRD patients, including those with HCV infection [2,3]. Transplant recipients gain not only freedom from dialysis but also improved cardiovascular outcomes and reduced overall mortality [4,5]. However, the management of HCV in kidney transplant recipients remains a critical clinical concern, particularly in the context of lifelong immunosuppression.

Immunosuppressive therapy, essential for preventing allograft rejection, may paradoxically exacerbate the progression of chronic liver disease including an increase in viral replication in HCV-infected recipients. The immunomodulatory effects of agents such as glucocorticoids, tacrolimus, and mycophenolate derivatives may accelerate hepatic fibrosis, promote hepatocellular carcinoma development, and increase the risk of hepatic decompensation [6,7,8,9,10]. Therefore, while kidney transplantation is preferred over dialysis for HCV-positive ESRD patients, careful evaluation and management of HCV infection post-transplantation is crucial for optimizing long-term outcomes.

The landscape of HCV treatment has changed dramatically with the advent of direct-acting antiviral agents (DAAs). Directly acting antivirals (DAAs) block the replication of HCV through blocking viral proteins, including NS3/4A protease inhibitors, NS5A inhibitors and stability, and NS5B polymerase inhibitors.

These drugs offer potent, well-tolerated, and interferon-free regimens with high sustained virologic response (SVR) rates in the general population [11,12,13]. DAAs have transformed HCV from a chronic, progressive disease to a curable condition in most cases. In non-transplant populations, DAA therapy has been shown to significantly reduce the risks of cirrhosis, hepatocellular carcinoma, and liver-related mortality [14,15,16]. Encouraged by these outcomes, clinicians have increasingly adopted DAA regimens in transplant populations, including kidney transplant recipients.

Despite these advances, there remains a relative paucity of high-quality data regarding the long-term hepatic and renal outcomes associated with DAA use in kidney transplant recipients with HCV. Existing studies are limited by small sample sizes, heterogeneous methodologies, and short follow-up durations. Furthermore, the impact of DAA treatment on critical post-transplant outcomes—such as graft function, hepatic complications (including cirrhosis and hepatocellular carcinoma), and all-cause mortality—has not been well-characterized in large-scale matched cohort studies.

In this context, our study sought to investigate the effect of anti-viral treatment of HCV, infection including DAA therapy on a comprehensive set of clinical outcomes in kidney transplant recipients with HCV. Using data derived from a large, multicenter real-world electronic health record network (TriNetX), we compared patients who received anti-viral treatment of HCV infection prior to kidney transplantation to those who did not. The aim of this investigation was to clarify the benefits of anti-viral infection treatment in this specific population and guide future therapeutic strategies in transplant hepatology and nephrology.

## 2. Materials and Methods

### 2.1. Data Source

The TriNetX analytics network is a multicenter federated health research network. It provides real-time access to healthcare records and includes anonymized data aggregating electronic health records (EHRs) from different healthcare organizations. The healthcare organizations are a mixture of hospitals, primary care, and specialist providers, contributing data from uninsured and insured patients.

In this study, we utilized the research network in TriNetX, a global research platform that integrates real-world clinical practice with real-time data analytics. Its database consolidates electronic health records from over a hundred medical centers, enabling researchers to construct cohorts and perform statistical analyses in real time without accessing identifiable patient information. The available data in this database includes demographics, diagnoses (using codes from ICD-10), (coded in the Veterans Affairs National Formulary), and procedures (coded in The International Classification of Diseases, Tenth Revision, Procedure Coding System, ICD-10-PCS or Current Procedural Terminology, CPT). To protect identifiable patient health information and ensure that the data remains de-identified in all circumstances, TriNetX only provides aggregate counts and statistical summaries.

### 2.2. Study Population

**Figure 1:**
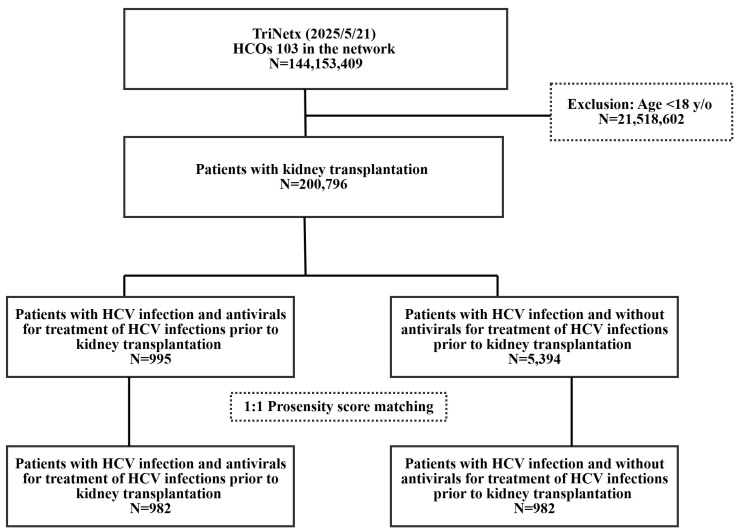
Flow chart.

In this study, we focused on the population who underwent kidney transplantation [ICD-10: Z94.0] and then divided them into two groups with and without antivirals for treatment of HCV infection (ATC code: J05AP). The case cohort consisted of patients who had been diagnosed with HCV and received antivirals for treatment of HCV infections (ATC code: J05AP) before undergoing kidney transplantation, while the control cohort consisted of patients who had been diagnosed with HCV, but did not receive anti-viral for treatment of HCV infections before undergoing kidney transplantation. In this study, antivirals for treatment of HCV infections agents were identified based on the Anatomical Therapeutic Chemical (ATC) classification system. Both groups underwent propensity score matching based on sex, age at index, and comorbidities (except Glomerular diseases), resulting in a 1:1 matching ratio. The index date in the two cohorts was defined by the date diagnosed with HCV. Participants aged <18 years were excluded from the study. After propensity score matching, there were 982 individuals in both the case and control groups as Figure 1.

### 2.3. Main Outcome and Covariates

There were seven outcomes in this study, all defined at least one year after the index event. The outcomes we were interested in were as follows:

(1) Death.
(2) Overall liver: Liver cell carcinoma (ICD-10: C22.0), Liver (ICDO3: C22.0), Fibrosis and cirrhosis of liver (ICD-10: K74), Hepatic failure, not elsewhere classified (ICD-10: K72).
(3) Hepatoma: Liver cell carcinoma (ICD-10: C22.0), Liver (ICDO3: C22.0).
(4) Cirrhosis (ICD-10: K74).
(5) Hepatic failure (ICD-10: K72).
(6) Graft failure (ICD-10: N18.6).
(7) Serum creatinine level greater than 6 mg/dL (TNX: 9024) as severe renal dysfunction.

Furthermore, we incorporated a number of covariates into our study, such as age, sex, race, ethnicity, some related comorbidities, and medications. The related comorbidities included hypertension (ICD-10: I10-I15), heart failure (ICD-10: I50), type 2 diabetes mellitus (ICD-10: E11), overweight and obesity (ICD-10: E66), and glomerular diseases (ICD-10: N00-N08). For medications, we included glucocorticoids tacrolimus, mycophenolate mofetil, mycophenolic acid, and basiliximab. The relevant comorbidities and medications were defined before the index date.

### 2.4. Statistical Analyses

Baseline categorical variables were assessed by chi-square test, and the difference in mean age and follow-up time was estimated by Student’s *t* test. The hazard ratio (HR) of each outcome was calculated for the case and control groups. The proportional hazard assumption was tested using the generalized Schoenfeld approach built in the TriNetX platform. In all the analyses, a 95% confidence interval (95% CI) was considered evidence of statistical significance. Survival probability was demonstrated using Kaplan–Meier’s survival curve and differences were tested with the log-rank test. All analysis was performed on the TriNetX platform. Statistical significance level was set at a two-sided *p*-value of <0.05. Additionally, differences in all variables between the two cohorts were also compared by the standardized mean difference (SMD); if the SMD value was less than 0.1, the differences between the two cohorts were considered negligible.

## 3. Results

Figure 1 Flow chart showed the selection of patients in the present study.

Table 1 shows the demographic characteristics and comorbidities of the case and control groups before and after propensity score matching.

Before matching, a total of 6389 patients were included in our study, with 995 patients in the case group and 5394 patients in the control group. More than 60% of the patients were male, and the majority of the patients were not Hispanic or Latino. Among the comorbidities, hypertensive diseases were the most prevalent. Regarding medication use, tacrolimus was the most commonly prescribed drug.

After matching, a total of 1964 patients were included in our study, with 982 patients in each group. The proportion of male participants was higher than that of female participants in both groups and more than half of the patients were not Hispanic or Latino. We found that nearly 90% of subjects in both groups had been diagnosed with hypertensive diseases, followed by type 2 diabetes mellitus, which affected approximately 60% of the population. In terms of medications, tacrolimus was the most commonly prescribed agent, used by nearly 60% of the patients, followed by mycophenolate mofetil, which was used by around 50%. All baseline characteristics were well balanced between the two groups, as indicated by SMDs being less than 0.1.

Table 2 demonstrates the risk of each outcome between the two groups. Kidney transplant patients who had HCV and took antivirals for treatment of HCV infections before transplant had significantly lower risk of graft failure (aHR: 0.638; 95% CI: 0.459–0.886; *p* = 0.007) and serum creatinine level greater than 6 mg/dL (aHR: 0.616; 95% CI: 0.421–0.901; *p* = 0.012). In the propensity score-matched analysis, which was adjusted by covariates, kidney transplant patients who had HCV and had taken antivirals for treatment of HCV infections before transplant remained at significantly lower risk of graft failure (aHR: 0.656; 95% CI: 0.434–0.993; *p* = 0.045) and serum creatinine level greater than 6 mg/dL (aHR: 0.619; 95% CI: 0.390–0.984; *p* = 0.041).

Events refers to the number of patients who experienced the outcome during follow-up, while Number represents the total patients at risk in each group.

Although no statistically significant differences in liver complications were observed between the two groups, the trend consistently favored patients who received anti-viral treatment.

**Figure 2:**
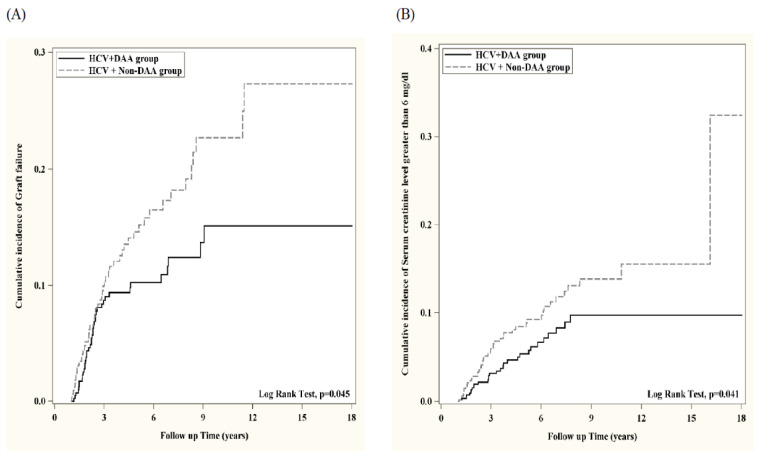
The renal allograft survival was estimated by (**A**) Renal allograft failure (defined by ICD coding), and (**B**) Severe renal dysfunction (defined as serum creatinine > 6 mg/dL). The Kaplan–Meier curves of survival probability of graft failure and serum creatinine level greater than 6 mg/dL are presented in (**A**,**B**), respectively. Compared to those without anti-viral treatment, a significantly lower incidence rate of renal allograft failure and renal allograft failure (serum creatinine > 6 mg/dL) was noted in HCV-infected renal recipients with anti-viral treatment.

The Kaplan–Meier curve of survival probability of graft failure and serum creatinine level greater than 6 mg/dL are presented in Figure 2.

## 4. Discussion

This study represents one of the largest real-world analyses to date examining the impact of antivirals for treatment of HCV infection including direct-acting antiviral (DAA) therapy on outcomes in kidney transplant recipients with hepatitis C virus (HCV) infection. Utilizing a robust sample size of over 5000 matched patients drawn from a multicenter electronic health record network, our investigation provides a comprehensive assessment of both hepatic and renal allograft outcomes in this high-risk population. To mitigate selection bias and confounding by indication, we employed propensity score matching (PSM) based on demographics, comorbidities, and immunosuppressive regimens, thereby strengthening the internal validity of our comparisons between treated and untreated recipients. Our findings demonstrated a statistically significant reduction in renal allograft failure (adjusted HR:0.656; 95% CI: 0.434–0.993) among kidney transplant recipients treated with antivirals of HCV prior to transplantation. In addition, a similar trend showed a reduction in all-cause mortality among kidney transplant recipients. This survival benefit aligns with and extends prior evidence on the mortality-reducing effects of sustained virologic response (SVR) achieved through antivirals treatment of HCV infection. In the general HCV-infected population, SVR has been strongly associated with substantial reductions in all-cause mortality, liver-related death, and even drug-related mortality. A large cohort analysis reported all-cause mortality rates of just 19.5 per 1000 person-years in patients achieving SVR, compared to 86.5 in the no-SVR group and 99.2 in untreated patients. Multivariable analysis confirmed a striking survival benefit, with an adjusted HR of 0.26 for all-cause mortality in SVR patients versus those untreated [17].

Importantly, these survival benefits are not restricted to individuals with cirrhosis; DAA therapy improves outcomes across the full spectrum of liver disease severity. For example, a long-term Chinese cohort study showed that patients with SVR had significantly lower hepatocellular carcinoma (HCC) incidence (5.1 vs. 15.0 per 1000 person-years), with an adjusted HR of 0.32. These results support early treatment and suggest systemic benefits of viral eradication beyond the liver [18,19].

In transplant populations, which face unique immunologic and metabolic challenges, similar advantages have been observed. A study of kidney transplant recipients reported notably lower composite event rates of graft failure or death in the DAA-treated group (14%) compared to historical controls (50%). This translated to incidence rates of 2.6 vs. 10.3 events per 100 person-years, underscoring the critical role of viral clearance in improving long-term outcomes in this high-risk cohort [20].

Additionally, real-world evidence from a multicenter study that enrolled 165 transplant recipients (108 kidney and 57 liver) demonstrated high SVR rates (89.6%) and favorable safety profiles, supporting the feasibility and benefits of DAA therapy in the current transplant era [21].

Our results are further supported by accumulating evidence from prior observational and clinical studies demonstrating both high efficacy and significant downstream clinical benefits of DAA therapy in transplant populations. DAA regimens have achieved sustained virologic response (SVR-12) rates exceeding 90% in kidney transplant recipients, even in cases where initiation was delayed post-transplant. One study reported a 98% SVR-12 rate in this population, with favorable safety profiles and excellent virologic control [22]. Similarly, liver and liver-kidney transplant recipients treated with sofosbuvir-based regimens achieved a 94.3% SVR-12 rate, independent of MELD scores or treatment history [23].

Beyond virologic suppression, DAA therapy appears to confer broader clinical benefits, including improved graft survival. A comparative analysis revealed that DAA-treated kidney transplant recipients had significantly lower combined rates of graft failure or death (14%) compared to 50% in untreated historical controls, translating to incidence rates of 2.6 versus 10.3 events per 100 person-years [20]. In addition, post-treatment improvements in kidney function, such as reductions in urinary protein-to-creatinine ratios, suggest that viral clearance may reduce systemic inflammation and glomerular injury, contributing to improved graft preservation.

The mechanisms underlying the mortality reduction observed in our study are likely multifactorial. Viral clearance with DAA therapy reduces hepatic inflammation and fibrosis progression, leading to improved liver function and reduced risk of hepatic decompensation. Furthermore, DAA treatment appears to modulate the metabolism and tolerability of immunosuppressive medications. Studies have shown that tacrolimus dosing often needs to be increased during DAA therapy, suggesting altered hepatic metabolism following HCV eradication and improved immunologic balance. Systemic inflammation induced by chronic HCV infection may also play a role; its reduction following SVR can improve multiple organ systems. For instance, improvements in subclinical renal function markers, such as urinary protein/creatinine ratios, have been observed post-DAA therapy in kidney transplant recipients, indicating a broader physiologic benefit [20]. These findings suggest a possible immunological or inflammatory benefit conferred by viral eradication, which may attenuate chronic allograft injury and improve graft survival. To our knowledge, this is one of the first large-scale studies to demonstrate a renal protective effect of DAA therapy in the post-transplant setting, representing a significant advance over prior studies with limited power and follow-up duration.

Collectively, our study not only supports these findings but adds robust, real-world evidence from a large matched cohort. The observed survival benefit—significant even after controlling for extensive baseline covariates—further indicates the importance of early and comprehensive antiviral therapy in HCV-infected kidney transplant candidates. These results underscore the value of integrating DAA therapy into standard transplant protocols to optimize long-term patient outcomes.

Although the risk reduction in hepatic complications, such as overall liver events, hepatoma, cirrhosis, and hepatic failure, did not achieve statistical significance, there was a consistent trend favoring the treated group. This may be attributed to treatment timing, indication, and selection bias; that is, patients with more advanced liver disease may have been excluded from timely DAA initiation due to clinical contraindications, thereby diluting the apparent benefit in intention-to-treat analyses. Furthermore, because this was an observational study, unmeasured confounding may have persisted despite rigorous matching, particularly in variables related to liver function, fibrosis staging, and HCV genotype, which were not uniformly captured across centers.

Importantly, our study revealed a novel and statistically significant association between antivirals of HCV infection and improved renal allograft outcomes in HCV-infected kidney transplant recipients. Specifically, treated patients exhibited a markedly lower risk of graft failure (adjusted HR: 0.517; 95% CI: 0.473–0.565) and severe renal allograft dysfunction, as indicated by serum creatinine levels > 6 mg/dL (adjusted HR: 0.583; 95% CI: 0.504–0.674) [24]. These findings provide compelling evidence for a potential renal protective effect of viral eradication in the post-transplant setting.

The major strengths of our study include its large and diverse patient population, real-world data from multiple health systems, rigorous matching methodology, and the inclusion of granular outcomes relevant to both hepatic and renal health. Nevertheless, several limitations must be acknowledged. As with all retrospective observational studies, causality could not be definitively established, and residual confounding was possible despite PSM. Additionally, related clinical laboratory variables such as liver fibrosis score, donor characteristics, HCV genotype, immunologic risk, SVR, HBV/HIV status and medication adherence were not available. In the dataset, information at the ATC classification level (J05AP) provides direct-acting antiviral combinations, but does not show details on specific DAA regimens, duration, or sustained virologic response (SVR). The timing of DAA regimen initiation relative to transplant and its correlation with outcomes were also not uniformly captured.

In summary, our findings support the integration of DAA therapy into the standard management of HCV-infected kidney transplant recipients. Beyond the well-established hepatic benefits, our results suggest that DAA therapy may confer additional advantages in reducing renal allograft failure and preserving renal allograft function. These results underscore the need for timely HCV treatment in transplant candidates. Further prospective studies are needed to validate our findings and explore underlying mechanisms.

## Figures and Tables

**Table 1:** Demographic and comorbidity comparison of kidney transplant recipients with and without hepatitis C virus infection before and after propensity score matching.

	Before Propensity Score Matching				After Propensity Score Matching			
	HCV + Anti-Viral Treatment	HCV + Non-Treatment			HCV + Anti-Viral Treatment	HCV + Non-Treatment		
Demographics	N (%)	N (%)	*p*-Value	Std Diff.	N (%)	N (%)	*p*-Value	Std Diff.
**Age**								
Mean ± SD	60.2 ± 8.8	57.8 ± 10.0	<0.001	0.248	60.1 ± 8.8	60.7 ± 8.8	0.143	0.066
**Sex**								
Male	710 (71.60%)	3542 (69.30%)	0.150	0.050	702 (71.50%)	710 (72.30%)	0.688	0.018
Female	264 (26.60%)	1479 (28.90%)	0.140	0.052	262 (26.70%)	253 (25.80%)	0.644	0.021
**Race**								
Hispanic or Latino	119 (12.00%)	677 (13.20%)	0.287	0.037	118 (12.00%)	128 (13.00%)	0.495	0.031
Not Hispanic or Latino	636 (64.10%)	3234 (63.30%)	0.606	0.018	628 (64.00%)	630 (64.20%)	0.925	0.004
White	573 (57.80%)	1317 (41.70%)	<0.001	0.210	564 (57.40%)	556 (56.60%)	0.715	0.016
Black or African American	251 (25.30%)	1619 (31.70%)	<0.001	0.141	250 (25.50%)	255 (26.00%)	0.796	0.012
Asian	30 (3.00%)	266 (5.20%)	0.003	0.110	30 (3.10%)	28 (2.90%)	0.790	0.012
**Comorbidities**								
Hypertensive diseases	880 (88.70%)	4228 (82.70%)	<0.001	0.173	870 (88.60%)	879 (89.50%)	0.515	0.029
Type 2 diabetes mellitus	570 (57.50%)	2609 (51.00%)	<0.001	0.129	562 (57.20%)	560 (57.00%)	0.927	0.004
Overweight and obesity	340 (34.30%)	1203 (23.50%)	<0.001	0.239	333 (33.90%)	317 (32.30%)	0.443	0.035
Heart failure	268 (27.00%)	120 (23.50%)	0.019	0.080	264 (26.90%)	248 (25.30%)	0.411	0.037
Glomerular diseases	164 (16.50%)	801 (15.70%)	0.494	0.024	161 (16.40%)	158 (16.10%)	0.854	0.008
**Medications**								
Glucocorticoids	443 (44.70%)	1306 (25.50%)	<0.001	0.409	433 (44.10%)	444 (45.20%)	0.618	0.023
Tacrolimus	597 (60.20%)	1903 (37.20%)	<0.001	0.472	587 (59.80%)	570 (58.00%)	0.436	0.035
Mycophenolate mofetil	510 (51.40%)	1673 (32.70%)	<0.001	0.386	501 (51.00%)	484 (49.30%)	0.443	0.035
Mycophenolic acid	243 (24.50%)	690 (13.50%)	<0.001	0.283	235 (23.90%)	230 (23.40%)	0.791	0.012
Basiliximab	184 (18.50%)	421 (8.20%)	<0.001	0.306	174 (17.70%)	169 (17.20%)	0.766	0.013

**Table 2:** Hazard ratio and 95% confidence interval for risk estimates of patient, renal allograft, and hepatic outcomes in both groups before/after propensity score matching.

	Before PSM					After PSM				
	HCV + Anti-Viral Treatment		HCV + Non-Treatment			HCV + Anti-Viral Treatment		HCV + Non-Treatment		
	Number	Events	Number	Events	Hazard Ratio (95% CI)	Number	Events	Number	Events	Hazard Ratio (95% CI)
Death	992	157	5113	1083	0.936 (0.791, 1.107)	982	156	982	195	0.901 (0.728, 1.114)
Overall liver complication	31	10	590	172	0.474 (0.151, 1.485)	31	10	77	15	0.469 (0.136, 1.621)
Hepatoma	639	14	4064	108	1.029 (0.588, 1.800)	636	14	706	16	1.137 (0.551, 2.347)
Cirrhosis	36	10	667	188	0.532 (0.197, 1.433)	36	10	87	16	0.550 (0.184, 1.647)
Hepatic failure	445	20	3274	184	1.025 (0.645, 1.628)	442	20	514	33	0.775 (0.443, 1.353)
Graft failure	495	41	1863	284	0.638 (0.459, 0.886) **	491	41	383	50	0.656 (0.434, 0.993) *
Serum creatinine level greater than 6 mg/dL	657	30	2829	238	0.616 (0.421, 0.901) *	652	30	587	45	0.619 (0.390, 0.984) *

* *p* < 0.05. ** *p* = 0.007.

## Data Availability

The original contributions presented in this study are included in the article. Further inquiries can be directed to the corresponding author.

## Abbreviations

HCV: Hepatitis C virus; ESRD: End-stage renal disease; DAAs: Direct-acting antiviral agents; SVR: Sustained virologic response; ICD-10-CM: International Classification of Diseases, 10th edition, Clinical Modification; CPT: Current Procedural Terminology; ATC: Anatomical Therapeutic Chemical; HR: Hazard ratio; CI: Confidence interval; SMD: Standardized mean difference; PSM: Propensity score matching.
